# Supplementary figures and images for: Spatial spillover effect of China’s tax and fee reduction policies on independent research and development evidence from dynamic Spatial Dubin analysis
Source: PLoS One. 2023 Aug 3;18(8):e0288535. doi: 10.1371/journal.pone.0288535 (PMC10399745; doi:10.1371/journal.pone.0288535)

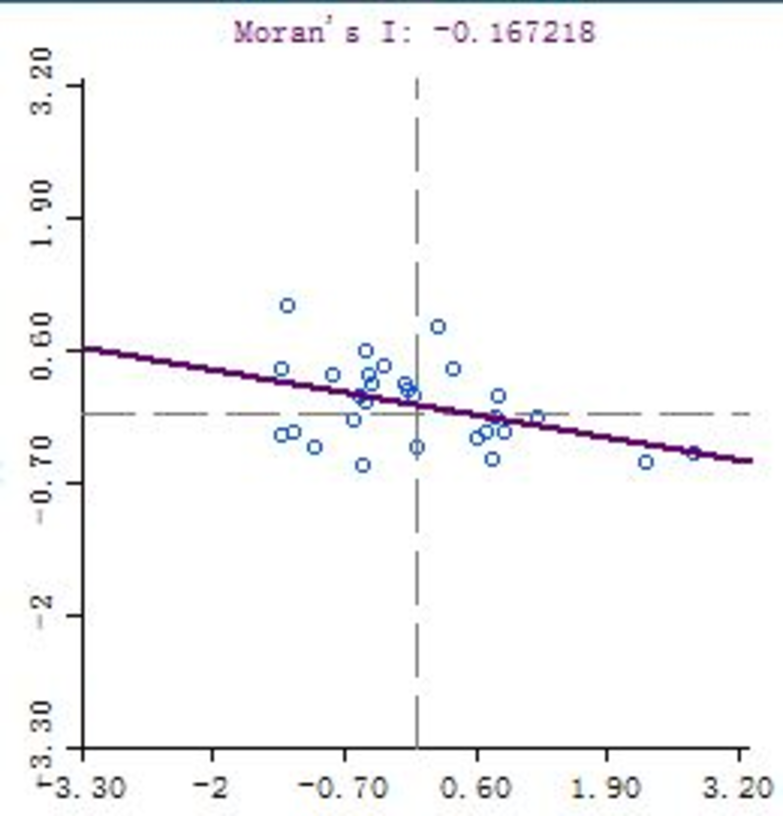

Supplement: S1 Raw images — (ZIP) [file pone.0288535.s002.zip › S2 1998-2019 moran‘I/year1998.tif]

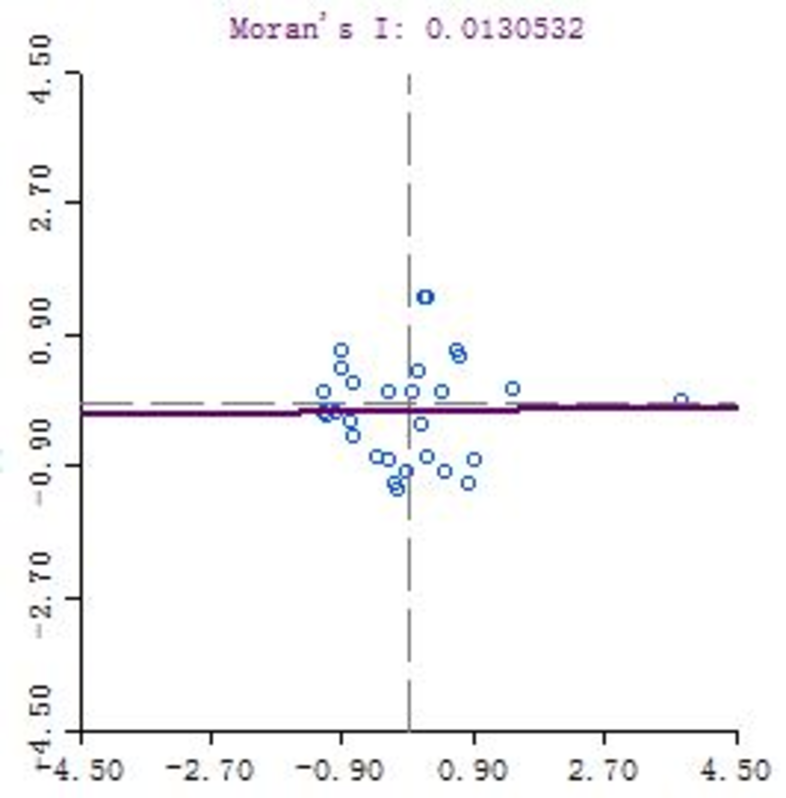

Supplement: S1 Raw images — (ZIP) [file pone.0288535.s002.zip › S2 1998-2019 moran‘I/year1999.tif]

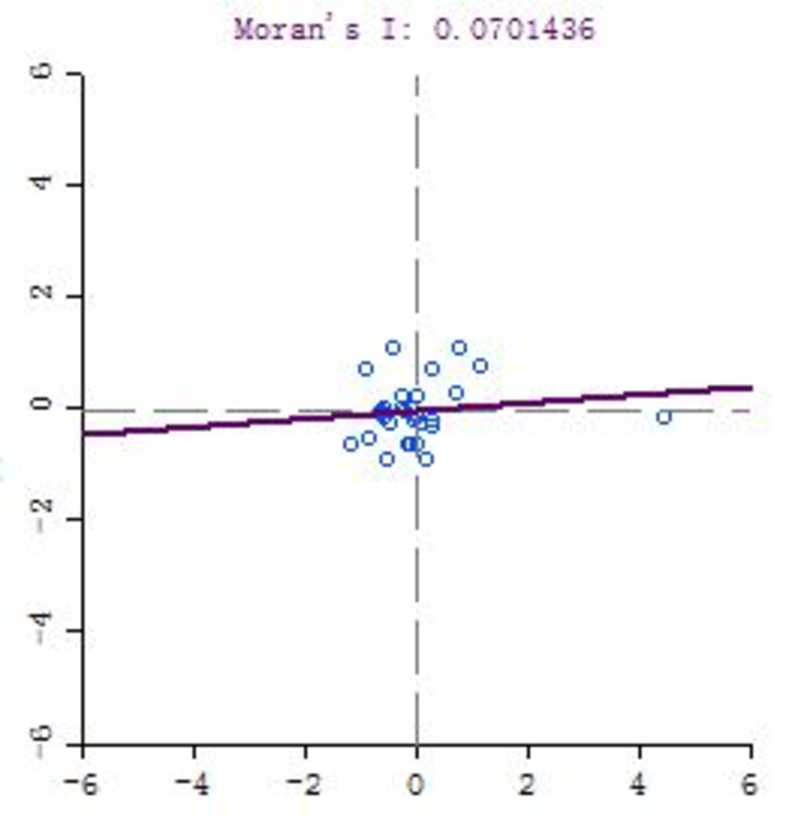

Supplement: S1 Raw images — (ZIP) [file pone.0288535.s002.zip › S2 1998-2019 moran‘I/year2000.tif]

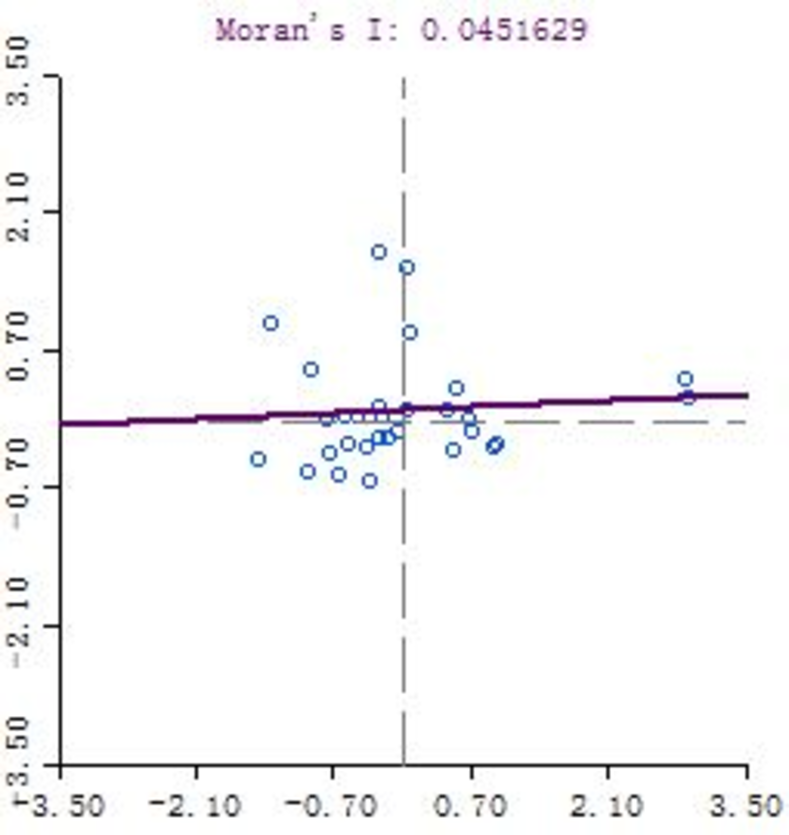

Supplement: S1 Raw images — (ZIP) [file pone.0288535.s002.zip › S2 1998-2019 moran‘I/year2001.tif]

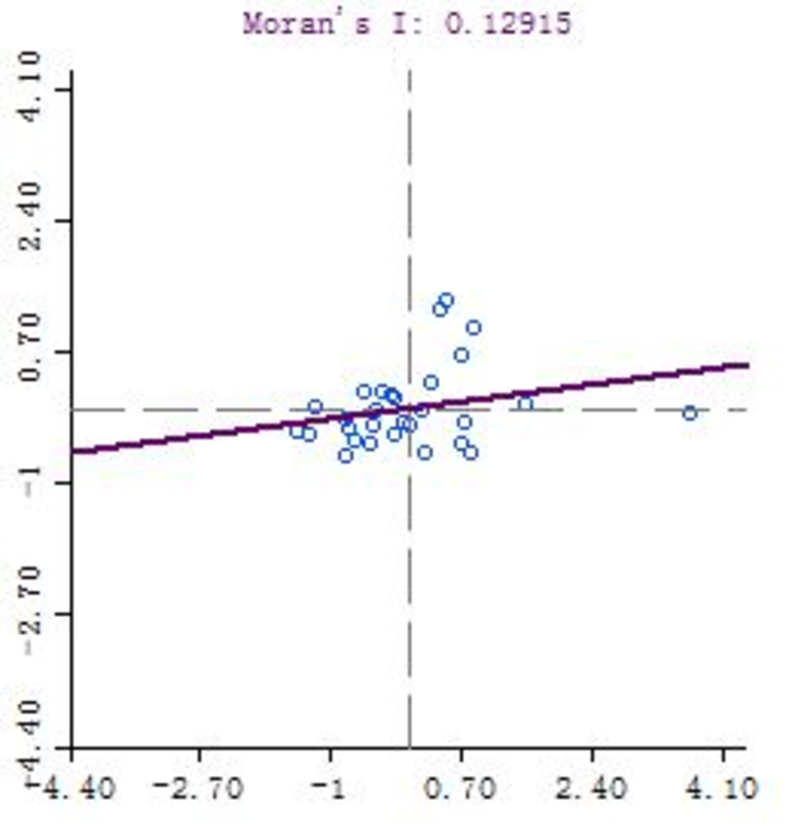

Supplement: S1 Raw images — (ZIP) [file pone.0288535.s002.zip › S2 1998-2019 moran‘I/year2002.tif]

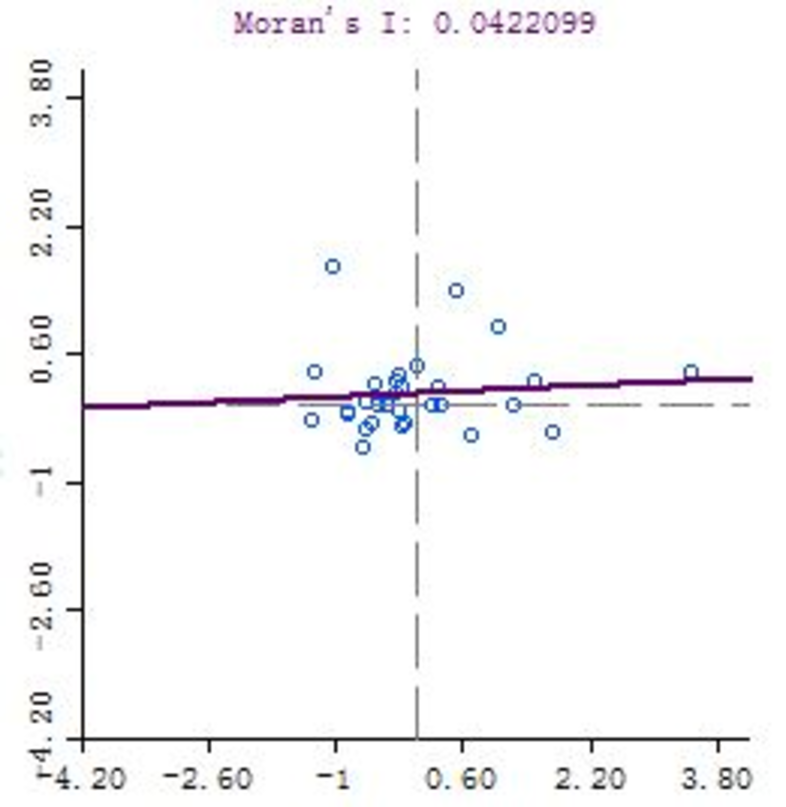

Supplement: S1 Raw images — (ZIP) [file pone.0288535.s002.zip › S2 1998-2019 moran‘I/year2003.tif]

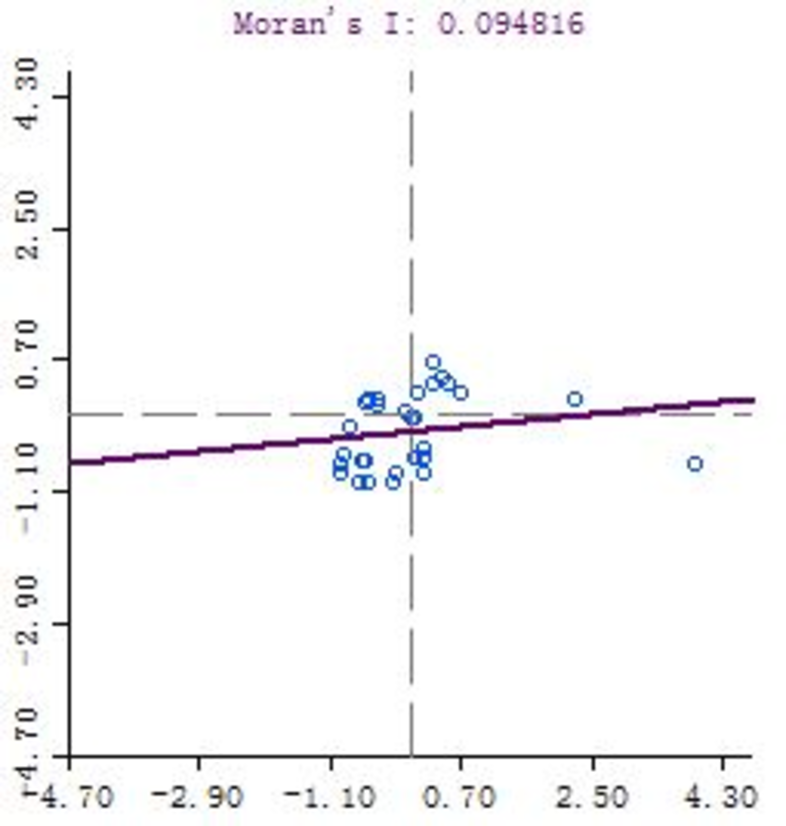

Supplement: S1 Raw images — (ZIP) [file pone.0288535.s002.zip › S2 1998-2019 moran‘I/year2004.tif]

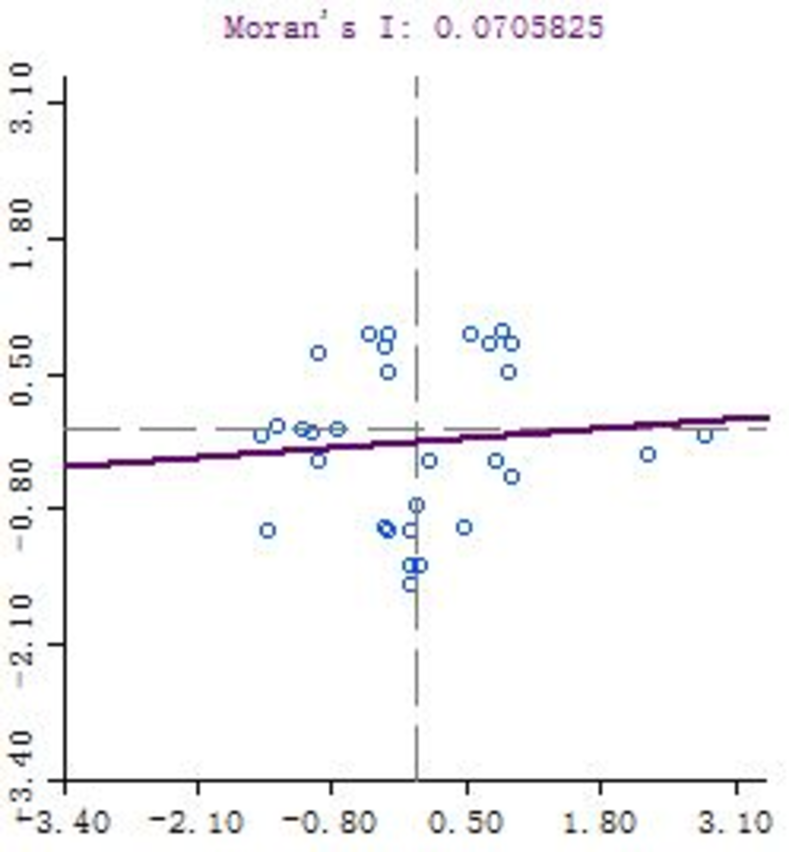

Supplement: S1 Raw images — (ZIP) [file pone.0288535.s002.zip › S2 1998-2019 moran‘I/year2005.tif]

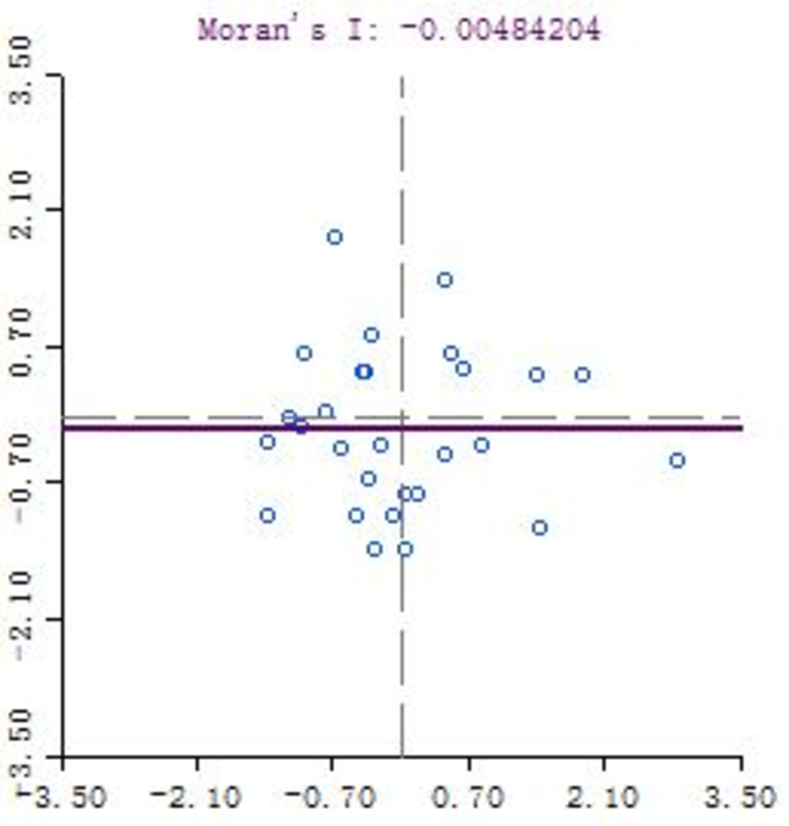

Supplement: S1 Raw images — (ZIP) [file pone.0288535.s002.zip › S2 1998-2019 moran‘I/year2006.tif]

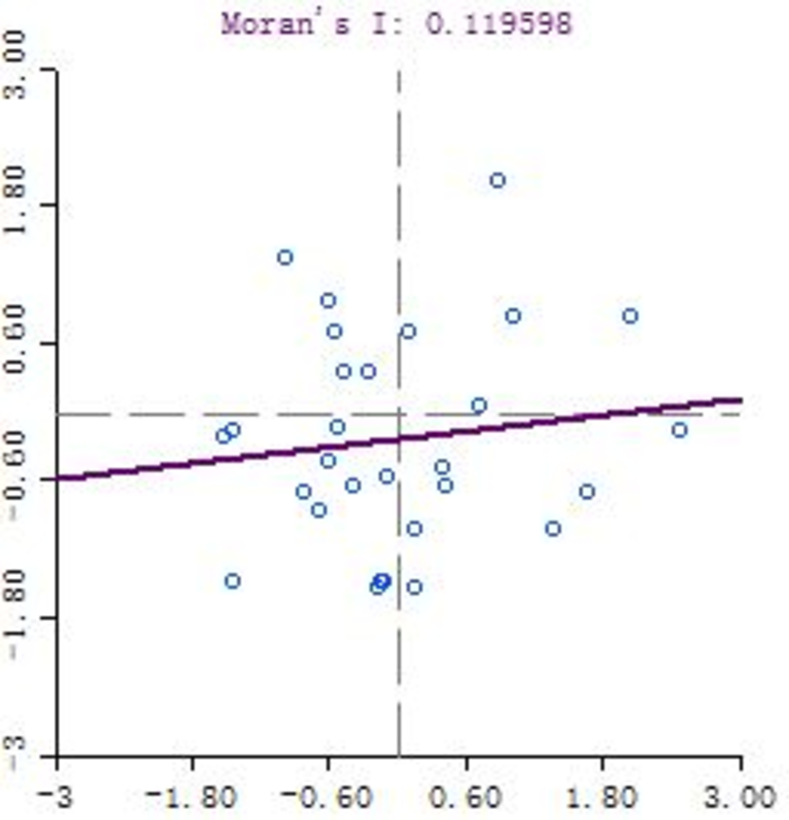

Supplement: S1 Raw images — (ZIP) [file pone.0288535.s002.zip › S2 1998-2019 moran‘I/year2007.tif]

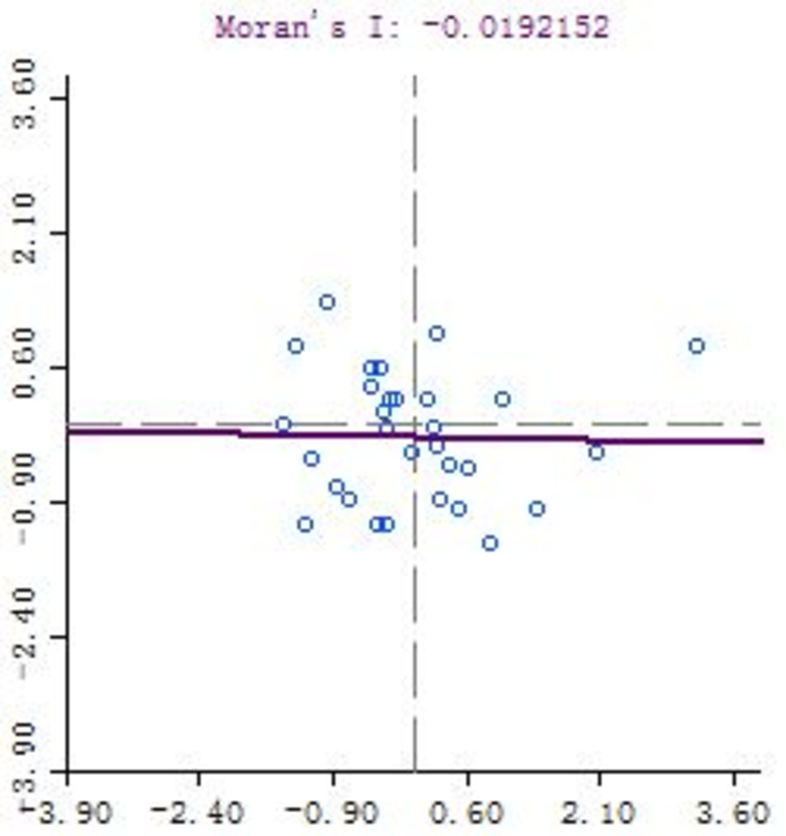

Supplement: S1 Raw images — (ZIP) [file pone.0288535.s002.zip › S2 1998-2019 moran‘I/year2008.tif]

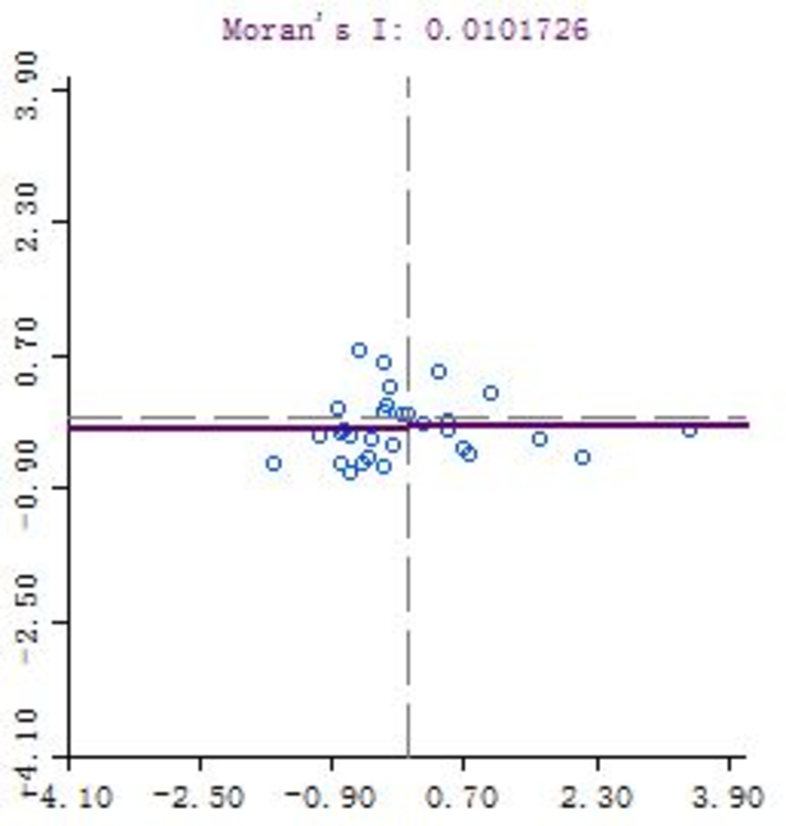

Supplement: S1 Raw images — (ZIP) [file pone.0288535.s002.zip › S2 1998-2019 moran‘I/year2009.tif]

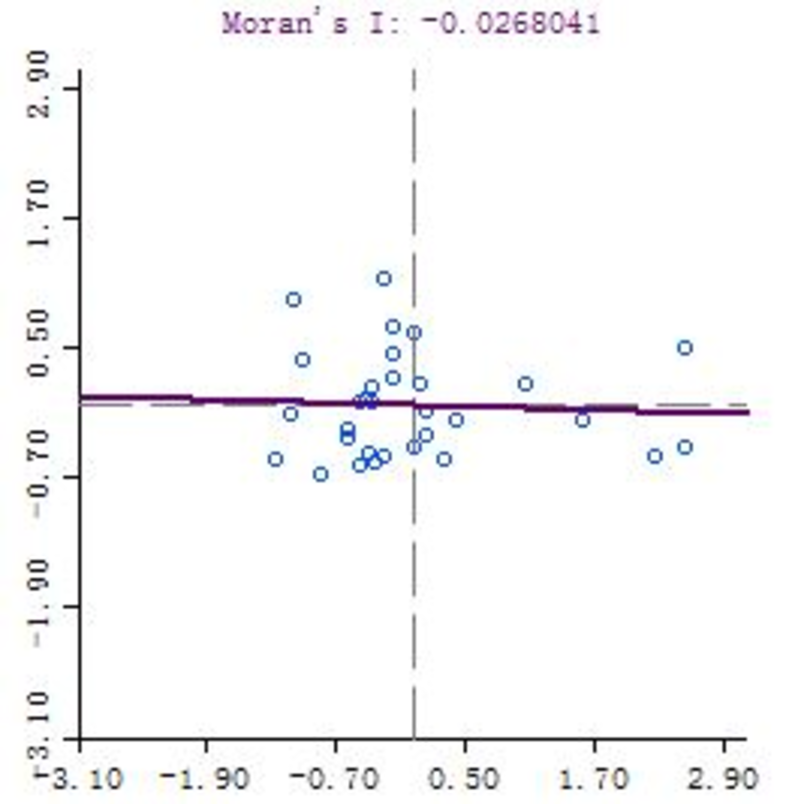

Supplement: S1 Raw images — (ZIP) [file pone.0288535.s002.zip › S2 1998-2019 moran‘I/year2010.tif]

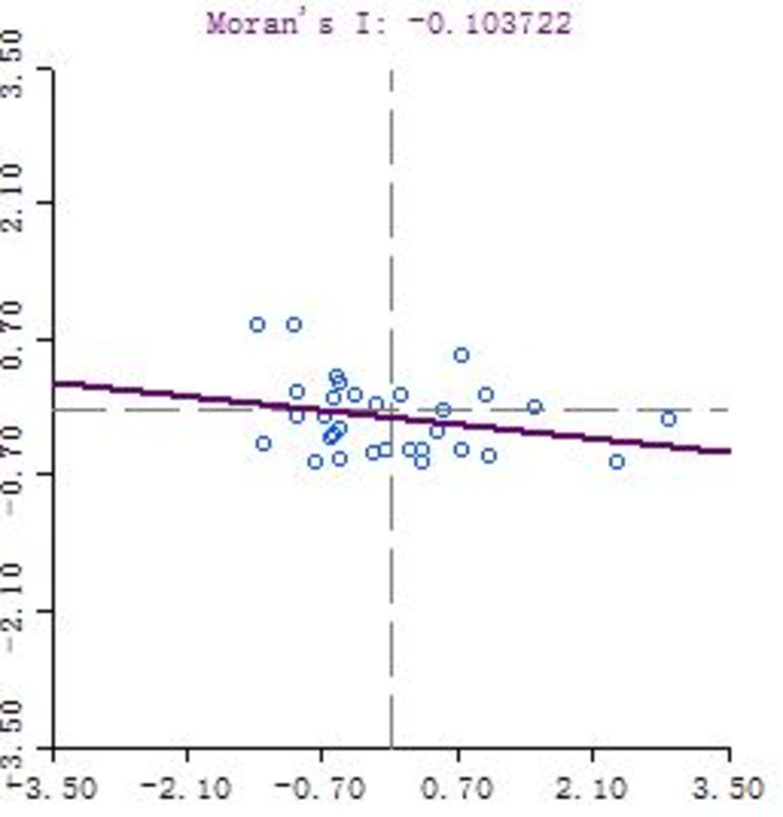

Supplement: S1 Raw images — (ZIP) [file pone.0288535.s002.zip › S2 1998-2019 moran‘I/year2011.tif]

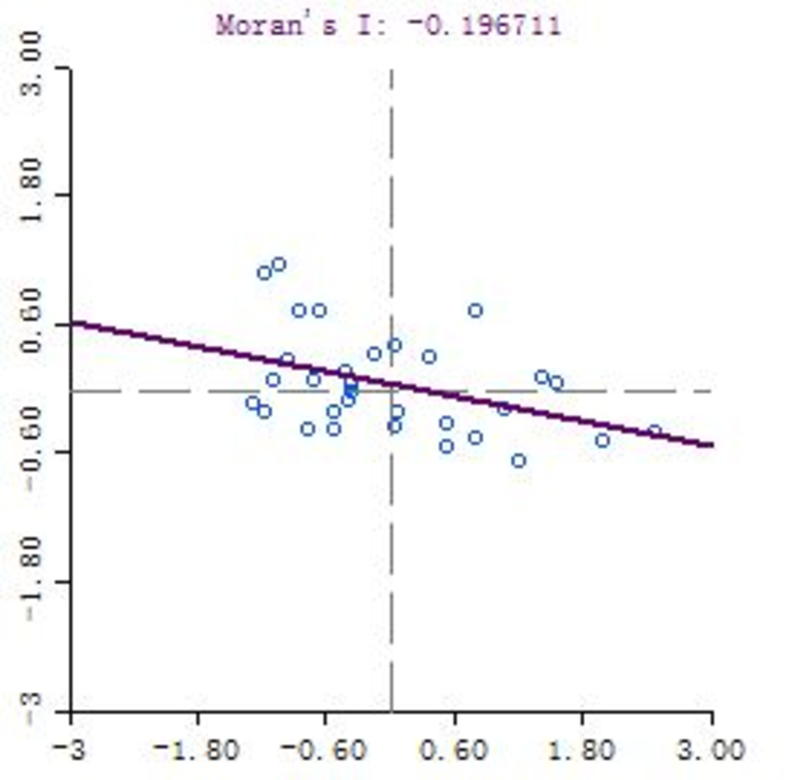

Supplement: S1 Raw images — (ZIP) [file pone.0288535.s002.zip › S2 1998-2019 moran‘I/year2012.tif]

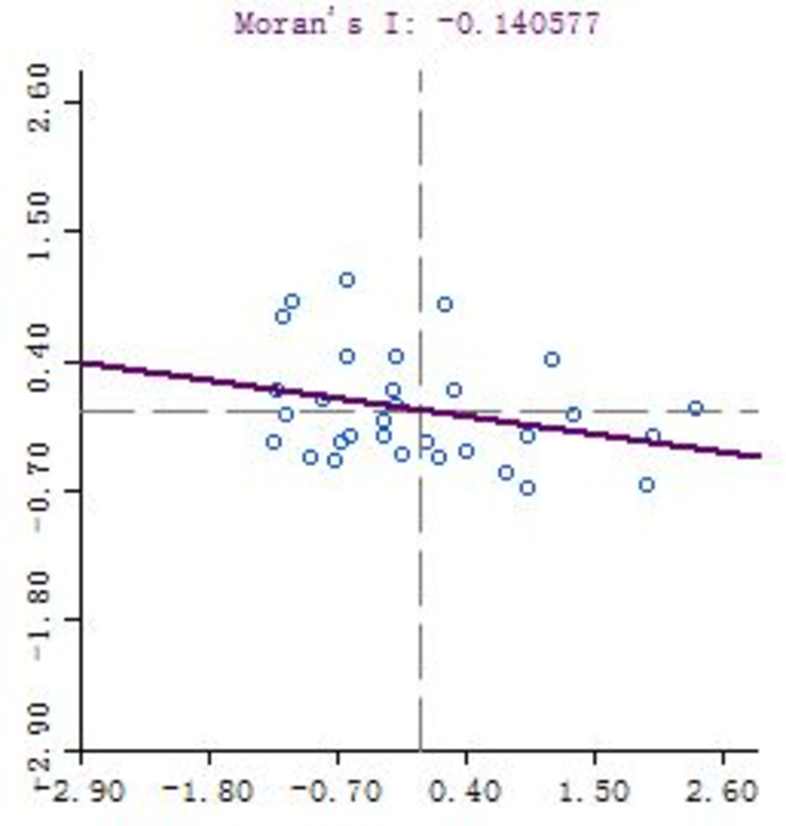

Supplement: S1 Raw images — (ZIP) [file pone.0288535.s002.zip › S2 1998-2019 moran‘I/year2013.tif]

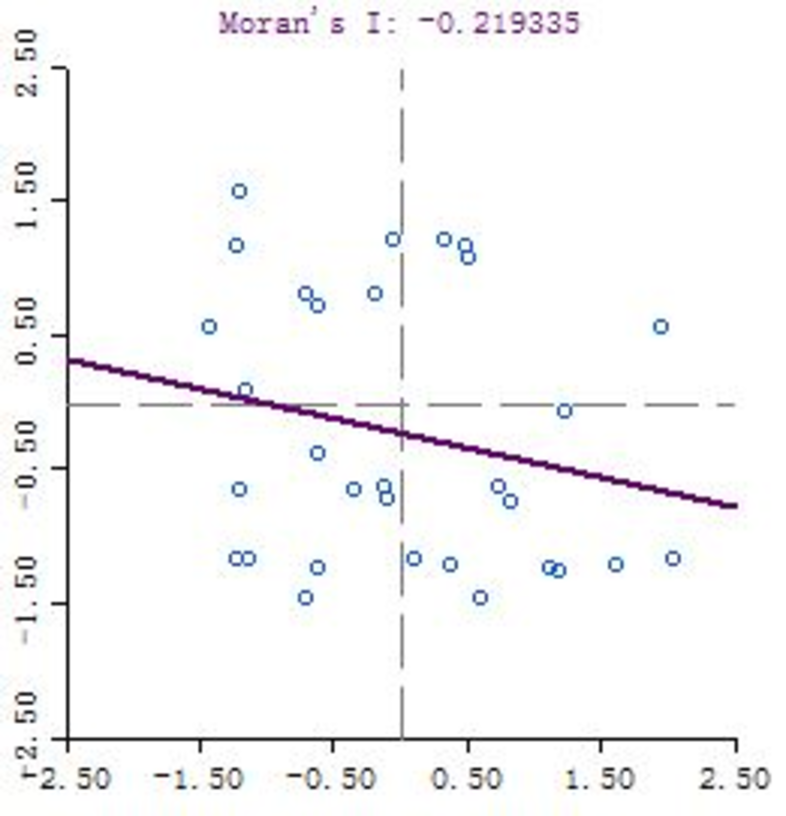

Supplement: S1 Raw images — (ZIP) [file pone.0288535.s002.zip › S2 1998-2019 moran‘I/year2014.tif]

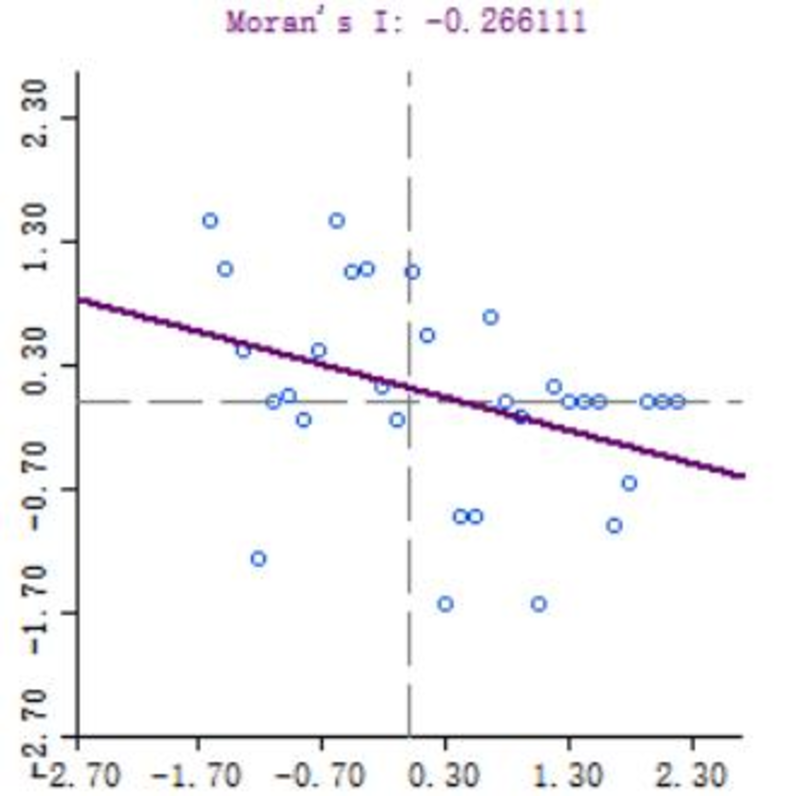

Supplement: S1 Raw images — (ZIP) [file pone.0288535.s002.zip › S2 1998-2019 moran‘I/year2015.tif]

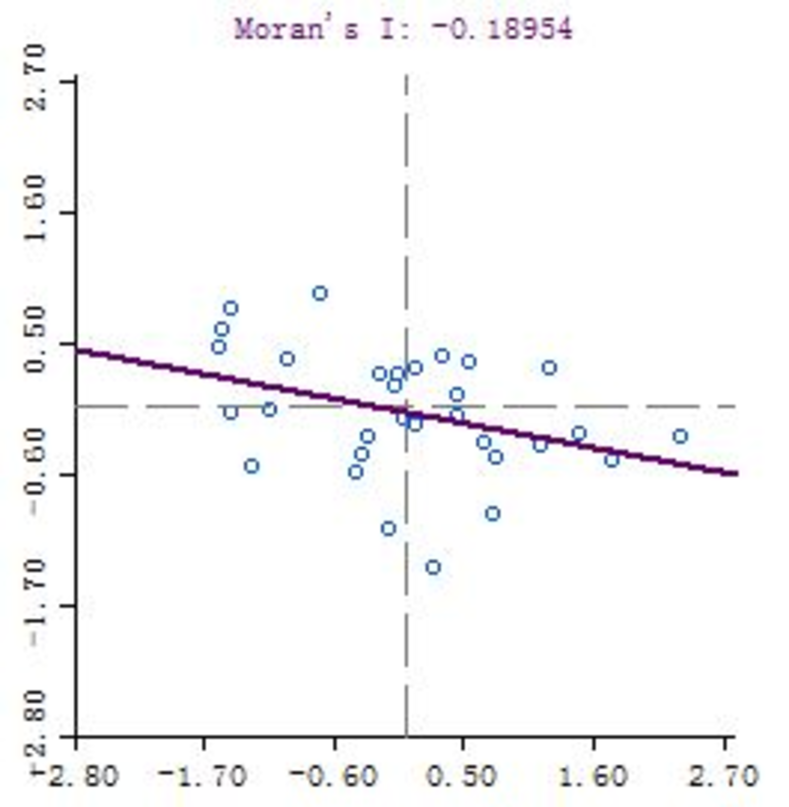

Supplement: S1 Raw images — (ZIP) [file pone.0288535.s002.zip › S2 1998-2019 moran‘I/year2016.tif]

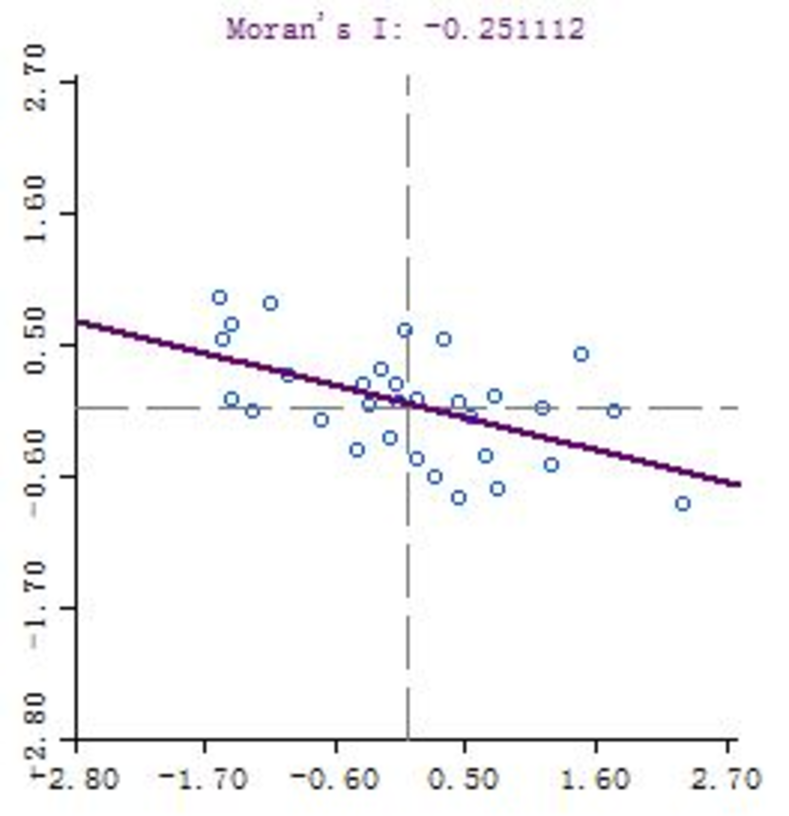

Supplement: S1 Raw images — (ZIP) [file pone.0288535.s002.zip › S2 1998-2019 moran‘I/year2017.tif]

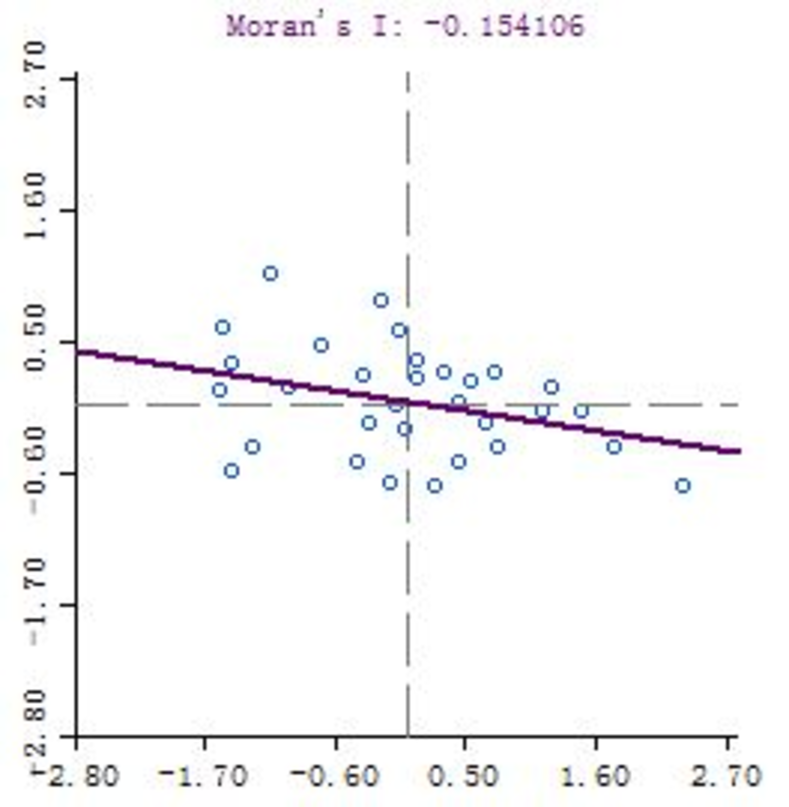

Supplement: S1 Raw images — (ZIP) [file pone.0288535.s002.zip › S2 1998-2019 moran‘I/year2018.tif]

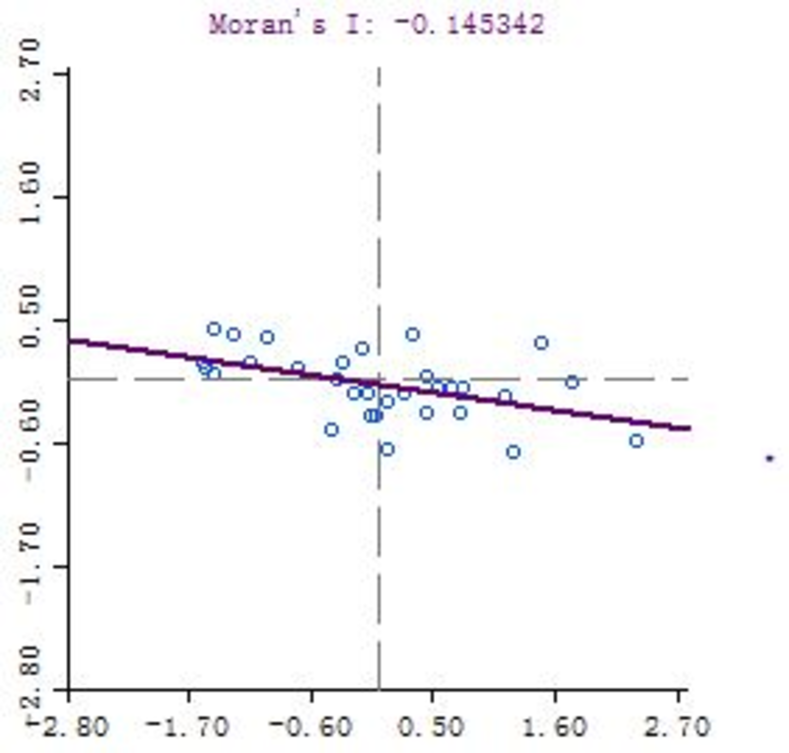

Supplement: S1 Raw images — (ZIP) [file pone.0288535.s002.zip › S2 1998-2019 moran‘I/year2019.tif]
