# Supplementary material for: Spatial spillover effect of China’s tax and fee reduction policies on independent research and development evidence from dynamic Spatial Dubin analysis
Source: PLoS One. 2023 Aug 3;18(8):e0288535. doi: 10.1371/journal.pone.0288535 (PMC10399745; doi:10.1371/journal.pone.0288535)
Supplement: Attachment — Submitted filename: Response to Reviewers.docx [file pone.0288535.s003.docx]

Dear reviewers and editors,

First of all, thank you very much for reading and revising my manuscript. Those comments are all valuable and very helpful for revising and improving our paper, as well as the important guiding significance to our researches. We have studied comments carefully and have made correction which we hope meet with approval. Revised portion are **marked in red** in the paper. The main corrections in the paper and the responds to the reviewer’s comments are as flowing:

1. **Comment:**

The abstract section is not well written. Authors should simply give the purpose of this paper, and mainly focus on the main findings of this paper.

**Response and revise:**

Many thanks to the reviewer for the comment of our abstract. Because some of the words in our article are relatively complex, the purpose and main findings of this study described in the abstract of our manuscript are really difficult to understand, which really causes confusion for reviewers and readers.

Here, we strongly agree with the reviewer. Here, we strongly agree with the reviewer's comment and we have reorganized the abstract content according to the principles requested by the review editor in order to give a simple and concise abstract so that the readers can understand the main purpose of our research and the content of the study. Please refer to the red content in the summary for details.

2. **Comment:**

This research is largely framed in the context of China. What about the research findings of other countries?

**Response and revise:**

Thanks for the prompt of the reviewer, the description of the research background of tax reduction and fee reduction, innovation and economic development in our manuscript really lacks the description of the background significance of other countries.

After discussion with my tutor, we very much agree with the reviewer's suggestion to add a description of the relevant research background of other countries in the manuscript. Due to space limitation, please see the red letter in the script for specific modifications in the introduction.

3. **Comment:**

The introduction section does not clearly introduce the research question，the main contributions of this paper and the differences with the other literature. This article lacks a description of innovation.

**Response and revise:**

Thanks for the guidance of the reviewer. After reading our manuscript, we found that we paid too much attention to the domestic innovation background. In view of this, we reorganize the introduction and add descriptions of research issues, main contributions, differences from other documents and innovations.

**4. Comment:**

What practical/professional and academic consequences will this study have for the future of scientific literature (theoretical contributions)?

Why is this study necessary? Again, the authors should make clear arguments to explain what is the originality and value of the proposed spatial spillover effect of China's tax and fee reduction on independent research in China. This should be stated in the final paragraphs of the introduction and conclusion sections.

In the first and second paragraphs of the introduction, the authors expressed an intention to understand the profile of spatial spillover effect, China's tax and fee reduction, independent research, and innovation performance. Also, bear in mind that contexts matter a lot unless contextual factor is taken into consideration.

The contribution of your work should be better highlighted. The introduction should outline:

(1) What is already known about the topic?

(2) What is not known about the subject and hence what does the study intend to examine. This means outline on what is the gap you seek to fill?

(3) What are the specific research questions the study focuses on?

**Response and revise:**

Thanks again for the detailed suggestions of the reviewer. After discussion with my tutor, we reorganized the literature in the introduction, supplemented the theoretical research, and described the necessity and the background and methods of tax reduction in other countries. On this basis, we summarized the research issues and the gaps filled. Please refer to the introduction of the article for details.

**5.Comment:**

The literature review was inadequate. I advise author to separate the literature review from Section 1. The research gap should be identified based on a more solid foundation.

The literature was not well organized. The literature review was inefficient. It can be organized in terms of different themes /research questions/ theories.

For instance, the author(s) listed many studies in the section of "theoretical background and hypotheses development". It is confusing. What are the exact theories employed in this study? Does the relevant literature correspond to the development of the four hypotheses?

This paper is an inaccurate representation of existing research gap. The literature cited in this paper is older and mostly non-international, which makes it difficult to check the authenticity of the cited ideas. The seminal literature should be cited.

**Response and revise:**

Thank the reviewer for reading the second part of our manuscript. We sincerely read the reviewer's suggestions. It may be the mistake in naming the original title of the second part that caused the reviewer to think that the second part is a literature review. In fact, the second part is the derivation of the econometric model. Unlike traditional research, which establish research hypotheses through literature - induction, summary and generalization. With the help of mathematical model, we use the constant effect function of substitution elasticity (CES) nested in Cobb Douglas (CD) function, and further introduce the elements of innovation and tax reduction policy to derive the research hypothesis through mathematical model deduction.

Our four assumptions are derived from the above mathematical model through derivation and differentiation. In addition, because CD production function and other basic mathematical theories are indeed put forward for a long time, on the basis of the mathematical model, we believe that we should still use the previous mathematical basis and introduce relevant elements on the basis of the theorem to derive. So reviewer will feel that the documents are outdated.

In order to avoid misunderstanding between reviewers and readers, we change the chapter name of Part II and briefly introduce the relevant introduction to the beginning of Part II.

**6. Comment:**

Variables are not enough sufficiently supported or clearly explained. So I advise authors to propose some suitable assumptions for your models following some literature review, which can make readers very clear about the source and basis of your assumptions.

**Response and revise:**

Thank the reviewer for the preciseness and meticulousness. We describe the variables that are not explained or less explained in strict accordance with the reviewer' suggestions. In addition, due to the problem of model description structure, the reasonable assumptions for the model have been elaborated in fact, but are relatively too complex. To avoid the doubts of readers and reviewer, the article adds a general description of the reasonable assumptions and basis of the model in "2.2 Spatial Durbin Model (SDM) method".

**7. Comment:**

The methodological contribution is good but they are similar to existing literature. I suggest authors provide explanations on what and why the methodology was applied different and valuable compared to existing methods. Why did not propose…….model. Please more interpretation.

**Response and revise:**

The reviewer's comments are very pertinent, and we very agree with the reviewer's suggestions. In order to enable readers to better understand the difference between the model used in the article and other models, and to understand the specific contribution and different values of the method, we have added a reasonable explanation of the spatial Dubin model (SDM) method, as well as the purpose and original intention of the method.

**8. Comment:**

I would like to see more discussion of the literature so that I can clearly identify the article relates to competing ideas. The discussion needs to be a coherent and cohesive set of arguments that take us beyond this study in particular and help us see the relevance of what the authors have proposed. The authors need to contextualize the findings in the literature and need to be explicit about the added value of your study towards that literature. Also, other studies should be cited to increase the theoretical background of each of the methods used. Findings should be contextualized in the literature and should be explicit about the added value of the study towards the literature. The contribution and implications of the article are yet to be specified. Please refer the style, 10.1016/j.jclepro.2022.132635.

**Response and revise:**

Thank you very much for reviewer’s professionalism. Literature is indeed an indispensable part of the thesis. Because there are a large number of mathematical model derivation in our paper, compared with traditional articles, it really appears that there are few documents. After discussion with our tutor, we tried to reorganize the literature, search and organize relevant research theories again. Limited by length, please refer to the revision of the full paper for details.

9. **Comment:**

Please check your submission in the review system. The Figures and equations are not suitable for publication. Please improve their quality. I advise author rewrite your equations using MathType or other professional equation tools.

Please present more figures and tables to illustrate your results.

**Response and revise:**

It is our negligence that the format problem is not adjusted properly. Thank the reviewer for reminding us. We use tools such as Math Type to adjust the format of the text in strict accordance with the reviewer's comments.

10. **Comment:**

Professional editing is required.

**Response and revise:**

Thank the reviewer for the suggestions. According to the suggestion of the reviewer, we invite our tutor and experts in relevant fields to put forward their opinions again and revise the article.

Finally, thank you again for your guidance, and thank you for reviewing and revising my revised paper again. I hope I can complete an excellent paper with your guidance and help. I sincerely hope my paper can be published in PLOS ONE.

Sincerely

Salute!

Meng Wu, Ruoyuan Sun
