# Supplementary material for: Spatial spillover effect of China’s tax and fee reduction policies on independent research and development evidence from dynamic Spatial Dubin analysis
Source: PLoS One. 2023 Aug 3;18(8):e0288535. doi: 10.1371/journal.pone.0288535 (PMC10399745; doi:10.1371/journal.pone.0288535)
Supplement: Attachment — Submitted filename: Response to Reviewers.docx [file pone.0288535.s004.docx]

Dear reviewers and editors,

First of all, thank you very much for reading and revising my manuscript. Those comments are all valuable and very helpful for revising and improving our paper, as well as the important guiding significance to our researches. My supervisor and I have made correction, which we hope meet with approval. Revised portion are marked in the paper. The main corrections in the paper and the responds to the reviewer’s comments are as flowing:

**Reviewer #1:**

1. **Comment:**

A concise and factual abstract is required. The abstract should state briefly the purpose of the research, the principal results and major conclusions. The abstract still needs to reorganize, especially the main findings of this study.

**Response and revise:**

Many thanks to the reviewer for the comment of our abstract. Because some of the words in our article are relatively complex, the purpose and main findings of this study described in the abstract of our manuscript are really difficult to understand, which really causes confusion for reviewers and readers.

Here, we strongly agree with the reviewer's comment and we have reorganized the abstract content according to the principles requested by the review editor in order to give a simple and concise abstract so that the readers can understand the main purpose of our research and the content of the study. Please refer to the red content in the summary for details.

2. **Comment:**

The authors spend a lot of time in the introduction section describing the background of the“innovation”, but do not clearly give the authors' motivation, main work and contribution.

**Response and revise:**

The reviewer's comments are very pertinent, and the text does tend to confuse the reader in the introduction, which provides less information about the full text. After discussion, we have reorganized the introduction based on the comments of the reviewers, simplified the background of "innovation", and further clarified the motivation, main work, and contributions of the research, so that the reviewers and readers can read it and know exactly what the paper will deal with.

After the revision, the introduction section of the article includes the domestic and foreign background, relevant research progress, as well as the motivation, main work, and contributions of this article. Due to space limitation, please see the track changes in the script for specific modifications.

3. **Comment:**

The key explanatory variable is questionable in the current draft. This paper use “the general public budget revenue” to measure “tax and fee reduction” is unconvincing. As this is the key explanatory variable, the authors should make 120% effort to cross-evaluate their findings for robust measures on “tax and fee reduction”, otherwise, the analysis does not make sense.

**Response and revise:**

Thanks for the reviewer's suggestion. We may have misunderstood the reviewer and readers due to our unclear description. In fact, we use the change in fiscal revenue as the core indicator for tax and fee reduction. If we rely on previous literature research and choose the changes in certain tax categories or expenses as the indicators for tax and fee reduction, it would be too one-sided, because the tax and fee reduction policy implemented in China is a macro level overall perspective reduction, not a single tax category. So, using the change in fiscal revenue as the core indicator, we can have a clear understanding of the overall momentum of tax and fee reductions. After all, the overall tax and fee reductions are clearly reflected in changes in fiscal revenue, including changes in tax and non tax revenue. Subsequently, we explained the selection range of fiscal revenue indicators in the change of fiscal revenue. Due to the ambiguity of the description, reviewers or readers may mistakenly believe that fiscal revenue is used to define tax and fee reduction.

Based on the above considerations, we have reorganized the description of the selection of core indicators, as detailed in the revised text.

**4. Comment:**

There is non-English language in the figures, e.g. Figure 1 and Figure 2. The author should correct them.

**Response and revise:**

Thanks to the reviewers for their rigor and meticulousness, which allowed us to re-examine our manuscript.

We followed the reviewers' suggestions and reorganized the content of the manuscript to remove non English language expressions.

**5.Comment:**

I would like the authors to polish their current draft towards better writing.

**Response and revise:**

Thanks to the reviewer, we have reorganized and polished the manuscript, and have invited our supervisor and relevant supervisors to review it, hoping to further improve our writing skills.Please review the modifications in the article for details.

**Reviewer #2:**

**1.Comment:**

A large number of professional terms are not used strictly enough, such as line 262, which should be Differences-in-Differences rather than double differences. And “breakpoint regression” “spatial metricis model” (line 270) and “spatial Dubin model” (line 30) are not correct also.

**Response and revise:**

Thanks for the reviewer's comment. We have reorganized the professional vocabulary in the entire text, especially the professional terminology related to spatial models. At the same time, adjust the capitalization of the terms and unify the writing style. It is worth discussing that in professional terminology, Durbin model can be named Durbin Model or Doberman Model.

For more details, please see the modifications in the text.

**2.Comment:**

The “Literature reviews” part seems weak, which required to improve and strengthen. I suggest to cite more latest researches in the relevant field to provide an up-to-date picture of work.

**Response and revise:**

The reviewer's comments are very pertinent. It should be noted that we did not write a separate chapter on literature review because China's implementation of tax and fee reductions is relatively short, and the effectiveness of tax and fee reduction policies often lags behind. The scope of policy impact is relatively broad, and most literature is only a partial impact, making it difficult to support a literature review on the entire mechanism of tax and fee reduction effects. We cited relatively new research in the first chapter and inserted relevant research literature into the entire text. Based on the current literature on tax and fee reduction in China, it has been basically covered. Perhaps there are many models in the literature, which has caused a slight hollowing out of the theory. After discussing with our supervisor, we have reorganized the literature and appropriately added some newer literature research.

For details,please see the red letter in the paper.

**3.Comment:**

Line 263: Maybe spatial DID model is better. It can not only realize the spatial correlation between variables, but also well evaluate the policy effect.

**Response and revise:**

The reviewer suggests that using a spatial DID model is indeed a good choice. In fact, before writing our paper, we tried the spatial DID model because it does have natural advantages in policy impact assessment models. Compared with traditional policy impact assessment methods, the DID model can largely avoid endogeneity issues, and setting virtual variables to determine whether policies occur can make estimates more scientific and accurate.

However, because China's tax and fee reduction policy gradually began after 2008 and has been implemented in some areas, the use of fixed time points to divide the treatment group and control group dummy variables is clearly inaccurate. If a multi-period DID model is adopted, firstly, it is difficult to ensure that the tax and fee reduction policies after 2008 can pass the parallel trend test, because there are many policies implemented in different production fields in China every year, and the tax and fee reduction involves too many fields to completely rule out the correlation effects of policies in various fields; Secondly, when we collect data to form a panel, due to sample data limitations, we are unable to fully cover all fields, and the annual group changes in each field cannot be tracked, making it impossible to use DID to evaluate the overall effect of tax and fee reduction policies; The third requirement is that DID meets exogeneity, and many policies in China provide a large amount of financial subsidies for independent innovation, which may lead to the impact of different policies entering the perturbation term, resulting in biased policy effects estimated by DID.

In addition, we have sorted out the relevant policies and measures for reducing taxes and fees.After the outbreak of the international financial crisis in 2008, China began implementing tax and fee reduction policies. The overall evolution of China's tax and fee reduction policies has gone through three stages:

During the period from 2008 to 2011, in response to the impact of the international financial crisis, especially when the export growth rate was negative 16% in 2019, macroeconomic policies were mainly based on demand management measures to respond to business cycle fluctuations. As far as positive fiscal policies were concerned, the focus was on driving economic growth through large-scale infrastructure construction marked by "four trillion yuan". The tax and fee reduction policies during this period were mainly structural tax reductions.

From 2012 to 2018, the tax and fee reduction policy, as an important component of the supply side structural reform "cost reduction" measures, played an increasingly important role in active fiscal policies. With the implementation of a series of policies such as the pilot and comprehensive implementation of the "business tax to value-added tax" reform, universal fee reduction, halving of income tax for small and micro enterprises, additional deduction of enterprise research and development expenses, and the combination of comprehensive and classified personal income tax, the scale of tax reduction and fee reduction continues to expand, and the macro tax burden level begins to decline.

Since 2019, in order to deal with the trade dispute between China and the United States and the impact of the COVID-19, on the basis of the tax and fee reductions of about 1 trillion yuan and 1.3 trillion yuan in 2017 and 2018, the scale of burden reduction of policies such as the larger scale tax and fee reductions implemented in 2019 and the large-scale rescue and assistance launched against the epidemic in 2020 has reached 2.36 trillion yuan and 2.6 trillion yuan respectively, and the scale of new tax and fee reductions in 2021 is about 1.1 trillion yuan.

In summary, spatial DID does have natural policy evaluation advantages, but neither single period DID nor multi period DID can adapt to the overall effect evaluation of tax and fee reduction policies. Therefore, we chose Spatial Durbin Model.

**4. Comment:**

Line 272-273: “Where the spatial weight matrix is added (the explanatory variable or the interpreted variable), it indicates which model this model is.” What does this sentence mean?

**Response and revise:**

Thanks to the reviewer for your careful and meticulous work. It is indeed our negligence that caused confusion for the reviewer and readers. Our original intention is to express that as long as the spatial weight matrix is selected, the form of the Spatial Durbin Model can be determined. There is indeed a problem with what we described in this paragraph.

After discussing with our supervisor, we have reorganized the content of this section and strive to correct any semantic ambiguity. Please see the text for modifications.

**5.Comment:**

It is confusing that does not clearly describe each explanatory variable and explained variable as “Section 2.3.1” shows.

**Response and revise:**

According to the suggestions of the reviewer, We do have a relatively simple description of this section, which has caused misunderstandings among the reviewers and readers. After discussing with our supervisor, we sorted out various variables and added descriptions of relevant variables to the paragraph text, such as “The degree of urbanization can represent the overall development level of a city, and can also be directly used to evaluate the economic development level and its derivative can partly represent the growth rate.”“The indicator used to measure the degree of openness of a country or province to the outside world can be foreign direct investment/GDP or import-export volume volume/GDP. We select foreign direct investment/GDP per capita and to prevent values from being difficult to observe, we list the original variables in Table 1”.

**6. Comment:**

Have other studies which support using DMSP/OLS night lighting data to describe the deviation of local fiscal competition strategy?

**Response and revise:**

Yes, there is research supporting the use of DMSP/OLS night lighting data to describe deviations in local fiscal competition strategies. There is a lot of research in related fields in China, such as drawing on the research of Zhu Deyun, Sun Ruoyuan, and Wang Bin (2019), considering the bias of local fiscal competition strategies under political promotion incentives, and referring to Liu Yanxu, Wu Wenheng, Wen Xiaojin, and Zhang Donghai (2013) using DMSP/OLS night light data to describe the bias of local fiscal competition strategies. Nighttime lighting data can to some extent display the construction of local infrastructure, which can reveal the competitive behavior of local governments towards infrastructure.

**7. Comment:**

Is the variable of “Independent research and development level” logarithmic?

**Response and revise:**

This is our negligence, as the description can easily lead to misunderstandings.Our original intention was to display the logarithmic data of the control variables. The previous reviewer asked us to display the data of the explained variable and the explanatory variable in Table 1 together. However, we overlooked that the control variables in the table were logarithmic and directly included the original data. Thanks to the reviewer for your careful and meticulous work. The logarithmic form of all variables used in the calculation of our article's model, so we have adjusted the data display format in the table to take logarithmic form.

**8. Comment:**

Line 447:IS “Spatial diagnostic test” a Spatial autocorrelation test? It should be explained.

**Response and revise:**

Spatial diagnostic test is a spatial autocorrelation test in our manuscript. Thanks for the reviewer's suggestion. The doubts of the reviewer are also the doubts of the readers. After discussing with our supervisor, we have changed the title of the chapter to "Spatial autocorrelation test" and added explanations on spatial autocorrelation testing. “Spatial autocorrelation statistics are a fundamental property used to measure geographic data: the degree of interdependence between data at a certain location and data at other locations. Usually, this dependency is called spatial dependency. Due to the influence of spatial interaction and diffusion, geographic data may no longer be independent of each other, but rather related. Spatial autocorrelation is an important starting point for the setting of spatial models. ”

**9.Comment:**

This paper has constructed three spatial weight matrices. The spatial autocorrelation test did not indicate which matrix was used.

**Response and revise:**

Thanks for the reviewer's suggestion. We supplement the description of the spatial weight matrix in the article."we use the Moran's I index for spatial autocorrelation test, where the spatial weight matrix selects the spatial adjacency matrix (0-1 matrix). The spatial weight of adjacent provinces is 1, and the spatial weight of non adjacent provinces is 0. The Moran's I index is an indicator used to measure spatial correlation, and its core is the spatial adjacency matrix".

**10.Comment:**

Line 471: The picture contains Chinese characters.

**Response and revise:**

Thanks to the reviewer for your diligence and attention. We changed Chinese characters to English according to the comments of the reviewer.

**11.Comment:**

Since this paper selects the dynamic spatial model in section 3.2, why does the Table 4 not include the coefficient of time lag?

**Response and revise:**

Thank the reviewer for the suggestions. We include spatial lag coefficients, and the spatial lag coefficients of the dependent variable are three: "time lag items of independent research and development", "W * independent research and development level", "W * time lag of independent research and development". W * policy effect is the product of four spatial weights and explanatory variables respectively. The meaning of spatial lag is different from that of time lag, and it is the impact of the surrounding area on the research area. In fact, this influence is the representation of the spatial weight matrix. In the dynamic spatial model, we selected four spatial weight matrices. Unlike what was estimated by the static spatial model, the static spatial model did not distinguish spatial weights, so we noted the spatial lag term, which is included in the W space weight. In addition, the theme of our manuscript does not involve the time lag of explanatory and control variables, as the time lag terms of explanatory and control variables do not affect the theme of this article. But the dynamic spatial model is the time lag of the dependent variable, which we have already included. We want to present the description of the variables as clearly as possible, as the characters may be too long, causing misunderstandings among the reviewer and readers.

**12.Comment:**

It should have regional heterogeneity or economic development heterogeneity analysis and empirical test?

**Response and revise:**

The reviewer studied the measurement method in depth and paid more attention to the robustness test of the estimator. In fact, in our manuscript, the spatial model adopts both static and dynamic models, including the selection of four spatial weights, which can already test the heterogeneity of regions and economies. Because adjacency matrix and geographical location matrix can verify regional heterogeneity, economic matrix and fiscal decentralization matrix are actually the embodiment of economic development heterogeneity. In addition, the introduction of light data is also a test of economic development heterogeneity to some extent.We have constructed a static model and a dynamic spatial model. The introduction of spatial and temporal parameters, as well as the spatial and time lag terms of the explained variables can overcome some endogeneity to a certain extent, and the decomposition of the coefficients of the Dynamic Spatial Durbin Model can more purely determine the marginal utility of the parameters, which has good persuasiveness for the subsequent estimation results of this paper. Furthermore, different spatial weight types were constructed, such as the adjacency weight matrix, geographical weight matrix, economic weight matrix, and fiscal decentralization weight matrix, which can improve the explanatory power of the parameters.

Perhaps our explanation of this section is not very clear, so we reorganized and explained the above question in the text to avoid confusion among reviewer and readers.

**13.Comment:**

In my opinion, adding policy background as a part will make the paper more complete.

**Response and revise:**

Thanks to the reviewer for the suggestion. As the reviewers pointed out, our article lacks a description of the policy background, which is indeed a bit abrupt. After discussing with our supervisor, we have added the policy background of tax reduction and fee reduction in the introduction. Due to space limitations, please see the modifications in the text for details.

“We have sorted out the relevant policies and measures for reducing taxes and fees. The overall evolution of China's tax and fee reduction policies has gone through three stages: During the period from 2008 to 2011, in response to the impact of the international financial crisis, especially when the export growth rate was negative 16% in 2019, macroeconomic policies were mainly based on demand management measures to respond to business cycle fluctuations. The tax and fee reduction policies during this period were mainly structural tax reductions; From 2012 to 2018, the tax and fee reduction policy, as an important component of the supply side structural reform "cost reduction" measures, played an increasingly important role in active fiscal policies. With the implementation of a series of policies such as the pilot and comprehensive implementation of the "business tax to value-added tax" reform, universal fee reduction, halving of income tax for small and micro enterprises, additional deduction of enterprise research and development expenses, and the combination of comprehensive and classified personal income tax, the scale of tax reduction and fee reduction continues to expand, and the macro tax burden level begins to decline; Since 2019, in order to deal with the trade dispute between China and the United States and the impact of the COVID-19, on the basis of the tax and fee reductions of about 1 trillion yuan and 1.3 trillion yuan in 2017 and 2018, the scale of burden reduction of policies such as the larger scale tax and fee reductions implemented in 2019 and the large-scale rescue and assistance launched against the epidemic in 2020 has reached 2.36 trillion yuan and 2.6 trillion yuan respectively, and the scale of new tax and fee reductions in 2021 is about 1.1 trillion yuan”.

**14.Comment:**

The conclusion is deficient. Authors should refine the important results and list them.

**Response and revise:**

Thanks to the reviewer for the insightful suggestions. We have sorted out the relevant writing process and conclusions based on the comments of the reviewers:

We first used Geoda to calculate the Moran's I index and plotted the corresponding Moran scatter plot for spatial correlation analysis. After verifying the spatiality, LM test and joint likelihood ratio LR test were used to estimate the LR and Wald values of the static spatial panel, and the Hausman model was selected. Furthermore, spatial models can be classified as estimates of Dynamic Spatial Durbin model. After partial differential decomposition of the coefficients of the Dynamic Spatial Durbin model, in order to make the estimation more robust, we further uses DMSP/OLS night light data and Malmquist productivity index to replace the relevant variables, and uses this to re-estimate from a more objective perspective. Although the data sample replacing the dependent variable is DMSP/OLS night light data, there is no significant heterogeneity in the estimation results in terms of significance and specific direction of action.

Our conclusion is that: whether using geographic matrices, economic matrices, or fiscal decentralization matrices for verification, local provinces are influenced by tax and fee reduction policies and tend to improve their own independent research and development levels in the short term, while neighboring provinces use them for technology purchase and dependence; Both direct and indirect effects are negative in the long run, indicating that tax and fee reduction policies not only fail to improve the local level of independent research and development in the long run, but also that in neighboring provinces; From a long-term and short-term perspective, local governments have strategic behaviors of political promotion incentives and independent innovation, rather than substantially improving the level of independent innovation; In addition, the externality of policy effects in the region also have a two-way effect of feedback.

**15.Comment:**

Further, authors should check the grammatical errors. I suggest authors to proof and edit the entire manuscript, and it will significantly help to improve English writing skills.

**Response and revise:**

Thanks for the reviewer's comment. After discussion with my supervisor, we combed the manuscript again, checked syntax error, and proofread the entire manuscript in strict accordance with the comments of the reviewer. We hope to improve our English writing skills through repeated revisions. Please see the red characters in the text for detailed modifications.

**Reviewer #3:**

**1. Comment:**

Please check your manuscript and improve its quality. The Figures and equations are not suitable for publication, especially Fig. 1 and Fig. 2.

**Response and revise:**

Thanks for the suggestions from the reviewer. We have strictly followed the reviewer’s advice and adjusted the figures and equations in the manuscript. Please see the manuscript for detailed revisions.

**2. Comment:**

Variables are not enough sufficiently supported or clearly explained in the manuscript. For example, "We tested the implementation effect of China's tax and fee reduction policies on independent innovation with the help of the dynamic spatial Dubin model (SDM), using DMSP/OLS night lighting data and Malmquist productivity index" in the abstract. However, the Malmquist productivity index is not explained in Section 2.3 Variables and data sources. Please explain.

**Response and revise:**

Thanks for the reviewer's suggestions. After discussing with our supervisor, we strictly followed the reviewer's suggestions and provided additional support and explanations in the manuscript. For the Malmquist productivity index, we add: “The Malmquist index was initially proposed by Malmquist in 1953, and Caves, Christensen, and Diewert began applying this index to the measurement of changes in production efficiency in 1982. The malquist index is a measure of the output input ratio, used to assess production efficiency and can be decomposed into several sub efficiency indicators. The calculation method of Malmquist index can be expressed by a formula: Malmquist index can be decomposed into technical progress index and technical efficiency change index. Among them, the technological progress index is the ratio of productivity indices at two time points, and the technological efficiency change index is the ratio of productivity indices at two time points divided by the technological progress index. The malmquist technology progress index is selected in this manuscript, and the specific calculation is: ”.

Finally, thank you again for your guidance, and thank you for reviewing and revising my revised paper again. I hope I can complete an excellent paper with your guidance and help. I sincerely hope my paper can be published in PLOS ONE.

Sincerely

Salute!

Wu Meng, Sun Ruoyuan
