# Supplementary material for: Spatial spillover effect of China’s tax and fee reduction policies on independent research and development evidence from dynamic Spatial Dubin analysis
Source: PLoS One. 2023 Aug 3;18(8):e0288535. doi: 10.1371/journal.pone.0288535 (PMC10399745; doi:10.1371/journal.pone.0288535)
Supplement: Attachment — Submitted filename: comments.docx [file pone.0288535.s005.docx]

The authors have addressed my concerns in their revision.The current version is much reliable compare to the previous draft. Still, I would like the authors to polish their current draft towards better writing.
