# Supplementary material for: Spatial spillover effect of China’s tax and fee reduction policies on independent research and development evidence from dynamic Spatial Dubin analysis
Source: PLoS One. 2023 Aug 3;18(8):e0288535. doi: 10.1371/journal.pone.0288535 (PMC10399745; doi:10.1371/journal.pone.0288535)
Supplement: Attachment — Submitted filename: Response to Reviewers.docx [file pone.0288535.s006.docx]

Dear reviewers and editors,

Thanks very much for the comments from the editor and reviewers. We have organized the description and grammar of the manuscript to improve our English writing skills.The main corrections in the paper and the responds to the reviewer’s comments are as flowing:

**Reviewer #2:**

1. **Comment:**

The explanations of the explained and explanatory variables are confusing.

**Response and revise:**

Many thanks to the reviewer for the comment. We originally intended to provide a detailed explanation of the variables of taxes and fees reduction, as well as the alternative lighting data variables used in robustness testing. The comment of the reviewer were indeed very relevant, and we also felt that the language expression was redundant and complex, which was confusing. Therefore, we reorganize the expression, remove redundant descriptions, and simplify the explanations of the dependent and explanatory variables as much as possible. Please refer to the revised manuscript for details.

2. **Comment:**

Figure 1 is not clear. Can it be replaced by a table? Or streamline it to a few years.

**Response and revise:**

Thanks to the reviewer for the suggestion. Indeed, the Moran's I Index image, which spans 22 years, cannot meet the clarity requirements with pixels. After discussing with our supervisor, we have presented the Moran's I Index chart for 4 years in the article, with images from other years attached. Please refer to the revised manuscript for details.

3. **Comment:**

Lack of description of the latest research trends.

**Response and revise:**

Thanks for the reviewer's suggestion. We strictly follow the reviewer's comment and provide a description of the latest research trends. Please refer to the revised manuscript for details.

**4. Comment:**

The author(s) do not adequately explain the reasons for not using spatial DID.

**Response and revise:**

Thanks to the reviewers for their rigor and meticulousness, which allowed us to re-examine our manuscript.

As suggested by the reviewer, using a spatial DID model is indeed a good choice，because spatial DID does have natural policy evaluation advantages. But neither single-period DID nor multi-period DID can adapt to the overall taxes and fees reduction policy effect evaluation:

1. The single period DID cannot determine the grouping time point between the treatment group and the control group. China's taxes and fees reduction policies include numerous small policies, and the implementation time varies in different regions. The taxes and fees reduction policy gradually began after 2008, and pilot projects were adopted first, with some areas gradually expanding to the whole country. The time points for implementing taxes and fees reduction policies in different provinces are different, and the use of fixed time points to divide processing and control groups into dummy variables is clearly inaccurate.
2. If using a multi-period DID model:

(1) It is impossible to exclude the relevant effects of other policies promoting production. The taxes and fees reduction policy is difficult to pass the parallel trend test of DID, as there are various forms of production promotion policies in different provinces and fields. The taxes and fees reduction policy is only one of the policy means to promote production, and its coverage is wide, making it impossible to completely rule out the correlation effects of different policies;

(2) The exogenous nature of taxes and fees reduction policies cannot be guaranteed. DID requires meeting policy externalities, and apart from tax and fee reduction policies, many other policies in China have a significant impact on independent innovation, which may lead to the impact of different policies entering the perturbation term, leading to a two-way causal relationship, resulting in biased policy effects estimated by DID；

(3) The sample changes rapidly and cannot track all fields, resulting in the inability to use DID to evaluate the overall taxes and fees reduction policy effect. Due to the limitations of sample data and the inability to track the changes in grouping in various fields each year, for example, some cities have implemented tax cuts in the manufacturing industry this year, announced tax cuts in the education industry next year, and some regions have reduced taxes this year but not next year, making it impossible to use DID to evaluate the overall tax and fee reduction policy effect.

Finally, thank you again for your guidance, and thank you for reviewing and revising my revised paper again. I hope I can complete an excellent paper with your guidance and help. I sincerely hope my paper can be published in PLOS ONE.

Sincerely

Salute!

Wu Meng, Sun Ruoyuan
